# Efficacy of Fermented Brown Rice by *Aspergillus oryzae* (FBRA) in Ulcerative Colitis: A Double‐Blind Randomized Trial

**DOI:** 10.1002/jgh3.70476

**Published:** 2026-10-05

**Authors:** Hideki Ishikawa, Yoshinari Onishi, Keiko Kataoka, Yoshitaka Murakami, Michihiro Mutoh, Hiroshi Kobayashi

**Affiliations:** ^1^ Molecular‐Targeted Preventive Medicine Kyoto Prefectural University of Medicine Kyoto Japan; ^2^ Department of Molecular Bacteriology, Institute of Health Biosciences The University of Tokushima Graduate School Tokushima Japan; ^3^ Department of Medical Statistics Toho University Tokyo Japan; ^4^ Sapporo Cancer Seminar Sapporo Hokkaido Japan

**Keywords:** *Aspergillus oryzae*, double‐blind randomized clinical trial, fermented brown rice, ulcerative colitis

## Abstract

**Background:**

Ulcerative colitis (UC) is a diffuse, nonspecific inflammation of the colon of unknown cause. Basic and clinical studies suggest that UC may be ameliorated by dietary fiber intake. Fermented brown rice by *Aspergillus oryzae* (FBRA) is produced by fermenting brown rice and bran with *A. oryzae*. We aimed to evaluate the efficacy of FBRA in patients with UC by a double‐blind, randomized crossover trial.

**Materials and Methods:**

Patients were divided into two groups: Group A (administered FBRA for 14 weeks, followed by a washout period, and then administered nonfunctional foods (NF) for 14 weeks) and Group B (NF and then FBRA). The primary endpoint is the presence or absence of improvement in clinical activity index (CAI) scores before and after the intervention. The secondary endpoints are the pre‐ and postintervention changes in blood data indicating inflammation (e.g., high‐sensitivity C‐reactive protein (CRP)) and nutritional status (e.g., serum proteins), and the adverse events.

**Results:**

Fifty‐six patients consented to participate: 19 in Group A and 21 in Group B completed the trial. Improvement in the CAI scores was not observed with FBRA. In the ITT analysis of the blood data, however, high‐sensitivity CRP, erythrocyte sedimentation rate hourly, white blood cell count, serum protein level, and serum albumin level all tended to be improved from the preintervention level (improvement ratio 1.70; 95% CI 0.97–2.98). No serious adverse event was observed.

**Conclusion:**

There is an expectation that FBRA may be effective in improving inflammation in patients with UC. Therefore, a large‐scale validation study should be conducted in the future.

**Abbreviations**
CAI: clinical activity index
CRP: C‐reactive protein
DSS: dextran sulfate
ESR: erythrocyte sedimentation rate
FBRA: fermented brown rice by *Aspergillus oryzae*
NF: nonfunctional foods
RBC: red blood cells
UC: ulcerative colitis
WBC: white blood cells

## 1 Introduction

Ulcerative colitis (UC) is characterized by diffuse nonspecific colorectal inflammation in the mucosa, often forming erosions and ulcers, and is common among young people with unknown causes. UC is a common disease in Europe and the United States; however, the number of patients with UC has recently been increasing rapidly in Japan.

Research on promoting dietary fiber intake to improve UC symptoms is underway [1]. The hypothesized mechanisms for the improvement of UC with the intake of fiber‐rich foods are as follows: (i) the effect of dietary fiber‐derived short‐chain fatty acids, especially butyric acid, (ii) reduction of mucosal injury through improved bowel movements, (iii) mucosal proliferative effects of dietary fiber, (iv) effects of antioxidants in vegetables and grains, and (v) dilution of toxic substances in the stool.

Brown rice fermented by *Aspergillus oryzae* (FBRA) is a food made by fermenting brown rice and bran *with A. oryzae* and is rich in dietary fiber [2]. FBRA is consumed by many Japanese people as a “so‐called health food” at a daily intake of 21 g. FBRA possesses the following advantages: (i) high hemicellulose content, which is metabolized by intestinal bacteria into short‐chain fatty acids with mucous membrane repair properties; (ii) high dietary fiber content, which increases stool volume; (iii) high phytic acid content (myoinositol 6‐phosphate), which has antioxidant properties and cyclooxygenase‐2 suppression activity [3]; and (iv) many minerals necessary for the body.

Kataoka et al. previously administered FBRA to dextran sulfate (DSS)‐induced UC mouse models and found that it prevented the onset of experimental enteritis [4]. However, to date, no study has administered FBRA to patients with UC.

Therefore, we conducted a double‐blind, randomized crossover clinical trial to evaluate the efficacy of FBRA in UC.

## 2 Materials and Methods

### 2.1 Trial Design

This was a single‐center, double‐blind, randomized crossover trial. After providing consent to participate, the patients were randomly assigned to two groups, Group A and Group B using a block randomization method. The assignment factors were age (< 40 years and > 40 years), sex, and extent of disease (rectal, left‐sided, or total colitis type). Assignments were made using computer‐generated random numbers by a co‐researcher and a trial statistician and were masked to other researchers and participants.

Interventions were FBRA or nonfunctional food (NF). Group A received FBRA for 14 weeks followed by a 12‐week washout period and NF for 14 weeks; Group B received NF for 14 weeks followed by a 12‐week washout period and then FBRA for 14 weeks.

The registration period was from July 2006 to December 2006.

Data management for the clinical trial was handled by Medical Research Support Ltd.

### 2.2 Trial Population

Patients with UC who met the eligibility criteria and were currently attending Osaka Chuo Hospital were invited to participate, and those who provided consent were included in the study.

The eligibility criteria were male and female patients aged 20 years or older and diagnosed with UC. The degree of inflammation and extent of disease were not specified. The diagnosis of UC was made comprehensively according to clinical, endoscopic, and histological findings in accordance with practice guidelines.

The exclusion criteria were fulminant UC, oral intake difficulties, steroids or immunosuppressive drugs at the time of entry, history of allergy to brown rice, and those deemed unsuitable by the attending physician.

At the beginning of the study, patients were invited to participate using an explanatory document. When consent was obtained, a documented consent record was recorded.

The call for participation was for 7 months, from October 2006 to April 2007. This is an exploratory study. Considering the feasibility of the study, a target of 60 patients (30 in each group) was set.

### 2.3 Investigational Food

The test foods were FBRA and placebo NF. The test foods were fine granules, and the FBRA and NF were odorless and virtually indistinguishable in appearance (Figure 1).

**FIGURE 1:**
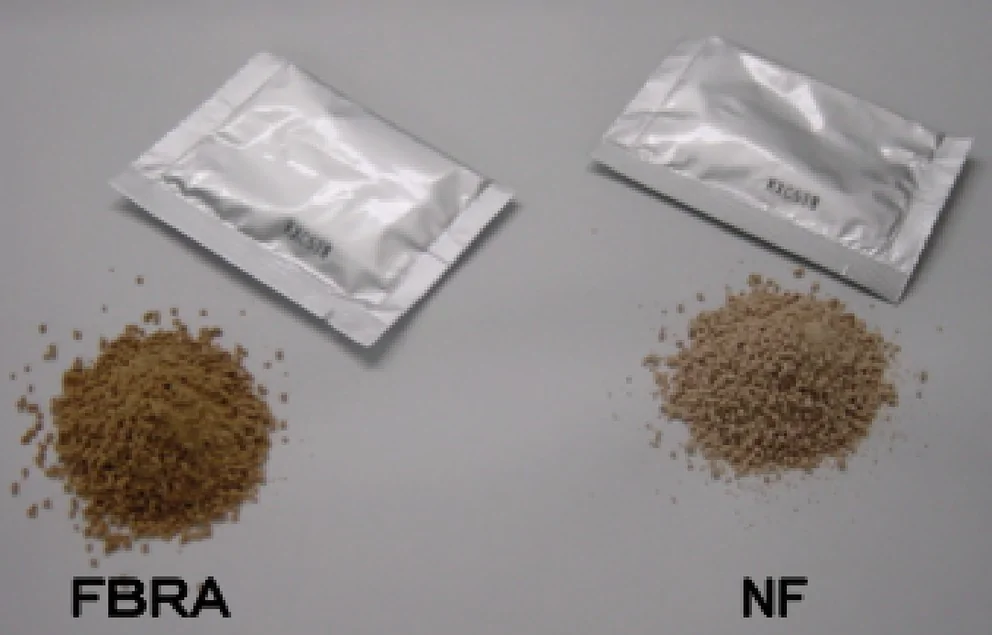
Aluminum packaging of FBRA and nonfunctional food (NF) and their appearances. The granules on the left show the appearance of FBRA and those on the right show the appearance of NF. Both were similar in shape and color.

The study was conducted as a double‐blind study, and whether the FBRA or the NF was not disclosed to patients and their physicians until after the completion of the study.

Group A received one packet of FBRA, and Group B received one packet of NF per day for the first week of treatment, three packets per day for the following week, and six packets per day for the following 12 weeks.

One packet contains 3.5 g of FBRA powder. Table 1 shows the composition measured at the start of the first and second halves of the administration.

**TABLE 1:** Nutritional composition of the test foods.

		FBRA		NF		Detection limit
		First half	Second half	First half	Second half	
Water	(g/100 g)	3.7	4.1	6.2	9.8	
Protein	(g/100 g)	17.0	16.2	8.9	8.4	
Fat	(g/100 g)	24.7	22.5	1.4	1.1	
Ash	(g/100 g)	11.6	10.5	0.5	0.4	
Carbohydrates	(g/100 g)	17.7	20.4	80.8	78.2	
Dietary fiber	(g/100 g)	25.3	26.3	2.2	2.1	
Energy	(kcal/100 g)	412	402	376	361	
Sodium	(mg/100 g)	9.5	10.3	3	2.8	
Phosphorus	(mg/100 g)	2.68	2.53	91.2	86.7	
Iron	(mg/100 g)	9.24	9.38	0.86	0.87	
Calcium	(mg/100 g)	49.5	51.1	10.3	9.5	
Potassium	(mg/100 g)	2.28	1.97	120	113	
Magnesium	(mg/100 g)	1.15	1.1	15.5	14.5	
Chlorine	(mg/100 g)	44	46	50	50	
Vitamin A (retinol equivalent)	(μg/100 g)	n.d.	n.d.	n.d.	n.d.	3 (μg/100 g)
Thiamine (vitamin B1)	(mg/100 g)	2.00	2.13	0.04	0.02	
Riboflavin (vitamin B2)	(mg/100 g)	1.01	1.08	0.03	0.03	
Vitamin B6	(μg/100 g)	3.51	3.53	47	37	
Total ascorbic acid (total vitamin C)	(mg/100 g)	n.d.	n.d.	n.d.	n.d.	1 (mg/100 g)
Vitamin E	(mg/100 g)	13.4	12.6	0.3	0.3	
Dietary fiber	(g/100 g)	21.6	22.6	3.6	3.9	
Water‐soluble indigestible polysaccharides	(g/100 g)	1.8	1.7	1.4	1.7	
Hemicellulose	(g/100 g)	9.5	10.3	1.7	1.6	
Cellulose	(g/100 g)	6	6.7	0.3	0.6	
Lignin	(g/100 g)	4.3	3.9	0.2	n.d.	0.2 (g/100 g)
Soluble dietary fiber	(g/100 g)	3.5	3.6	0.9	1	
Insoluble dietary fiber	(g/100 g)	21.7	23.4	1.1	1.2	
Total amount	(g/100 g)	25.2	27	2	2.2	
Phytic acid	(g/100 g)	7640	5930	89.3	76.2	
Bromine	ppm	2	2	n.d.	n.d.	1 ppm
BHC (organochlorine insecticides)	ppm	n.d.	n.d.	n.d.	n.d.	0.01 ppm
Aldrin	ppm	n.d.	n.d.	n.d.	n.d.	0.005 ppm
Endrin	ppm	n.d.	n.d.	n.d.	n.d.	0.005 ppm
Parathion	ppm	n.d.	n.d.	n.d.	n.d.	0.01 ppm

NF is a granule formulation of lightly roasted wheat flour with 15% starch, prepared using the same method as FBRA. One packet contains 3.5 g NF. Similar to FBRA, the composition measured at the beginning of the first and second halves of administration is shown in Table 1. B1, B2, E, phytic acid, and NF contained more phosphorus, potassium, magnesium, and vitamin B6 than the FBRA.

After the allocation, the test food (FBRA or NF) was administered for 14 weeks. Intake of the test food was monitored using a dose logbook.

### 2.4 Assessment

Those who agreed to participate were ascertained by the attending physician prior to allocation: clinical symptoms at entry, colonoscopy findings immediately prior to participation, treatment, and subjective symptoms using the clinical activity index (CAI) [5].

#### 2.4.1 Use CAI Score to Identify Subjective Symptoms

The participants were examined for items relevant to the CAI. The CAI scores, which have been shown to reflect changes in the clinical status of patients with UC, were based on the following:

- Number of episodes of diarrhea (0–4).
- Presence of nocturnal diarrhea (0–1).
- Degree of visible blood in the stool (0–3).
- Presence of fecal incontinence (0–1).
- Degree of abdominal pain or cramping (0–3).
- General well‐being (0–5).
- Degree of abdominal tenderness (0–3).
- Need for antidiarrheal drugs (0–1).

The overall CAI scores ranged from 0 to 21. The score increases as the symptoms worsen. Active stage is defined as more than five points. To evaluate improvements in CAI, pre‐ and postintervention scores were compared. A decrease was classified as an improvement, no change in the scores as stable, and an increase as a worsening.

Blood samples and confirmation of clinical symptoms will be conducted by visiting the hospital 14 weeks prior to administration and at the end of the first half of the study food administration, 26 weeks prior to the start of the second half of the study food administration, and 40 weeks at the end of the second half of the study food administration.

Blood samples were collected for the analysis of high‐sensitivity C‐reactive protein (CRP), erythrocyte sedimentation rate (ESR), blood count, and biochemical tests (aspartate transaminase (AST), alanine transaminase (ALT), alkaline phosphatase (ALP), lactate dehydrogenase (LDH), protein, albumin, gamma‐glutamyl transferase (gamma GPT), creatine kinase (CPK), total cholesterol, triglycerides, uric acid, blood urea nitrogen (BUN), and creatinine (Cr)). To evaluate improvements in blood biochemical test values and white blood cell count, pre‐ and postintervention values were compared. A decrease was classified as an improvement, no change in the values as stable, and an increase as a worsening.

### 2.5 Trial Endpoints

The primary endpoint was the percentage improvement in CAI after the first half of the study period. Secondary endpoints were changes in blood laboratory values indicative of inflammation and nutritional status (high‐sensitivity CRP, hourly blood sedimentation rate, white blood cell count, serum protein, and serum albumin), adherence to the study foods, and frequency of adverse events.

### 2.6 Statistical Analysis

The primary endpoint compared the proportion of patients whose CAI scores improved after the first half of the study food administration between the two groups. The *χ* ^2^ test will be used to calculate risk ratios and 95% CI. The main analysis will be performed using ITT according to the allocation results, regardless of the degree of intake of the test food.

Secondary endpoints were analyzed by comparing the two groups using chi‐square tests for categorical variables and *t*‐tests for continuous variables. The risk ratios and 95% CI were calculated for several items. PP analysis was performed for those who did not wish to discontinue consuming the study food and those who consumed the study food as scheduled. Intent‐to‐treat (ITT) and per‐protocol set (PPS) analyses were performed for the secondary endpoints.

## 3 Results

### 3.1 Participation Procedure

A flowchart of participation is shown in Figure 2. Seventy‐three patients were invited to participate; 17 refused and 56 provided their consent. After consent was obtained, patients were randomly assigned to two groups: 29 in Group A and 27 in Group B. Three patients in Group A and one patient in Group B refused to participate after assignment and before consuming the test food.

**FIGURE 2:**
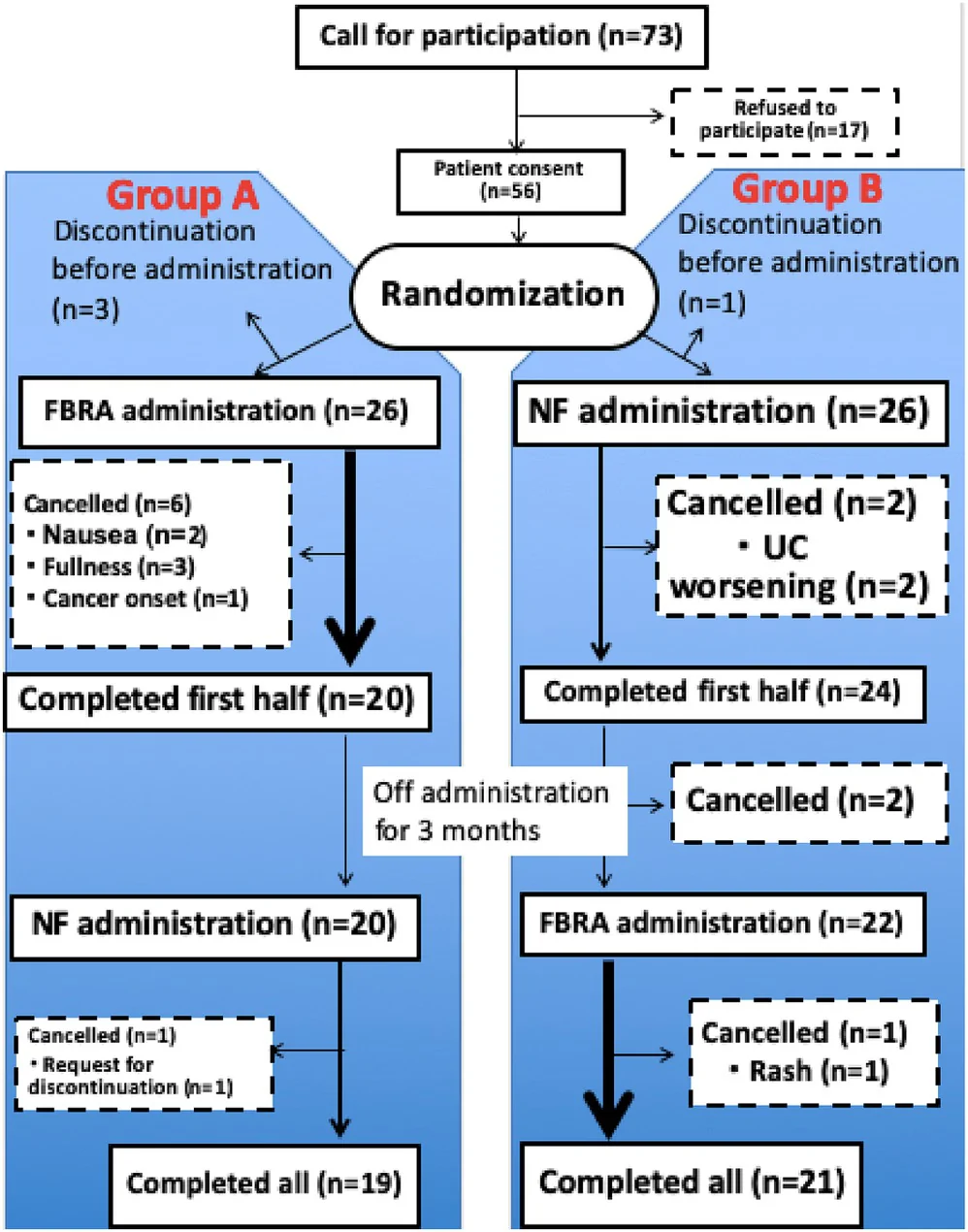
A flow chart of participation. After providing informed consent, the participants were randomly divided into Group A (left side) and Group B (right side). The number of participants and canceled numbers are shown in the solid and dotted frames, respectively.

In the first half of the study, 26 patients in Group A received FBRA and 26 patients in Group B received NF. Six (23%) of the 26 patients in Group A who received FBRA wanted to discontinue treatment because of nausea (two patients), abdominal fullness (three patients), and worsening cancer in other organs (one patient). Nausea and fullness resolved after the discontinuation of the test food. Two of the 26 patients in Group B who received NF wished to discontinue treatment because of worsening UC symptoms. Twenty patients in Group A and 24 patients in Group B completed the first 3 months of treatment. Those who wished to discontinue taking the drug continued to visit the hospital, and medical examinations and blood sampling were continued.

During the washout period of 3 months, two patients in Group B wished to discontinue the study. During the second half of the study, 20 patients in Group A received NF, 22 in Group B received FBRA, and one patient in Group A wished to discontinue; one patient in Group B developed a rash and discontinued the drug. During the entire study period, 19 patients in Group A and 21 in Group B did not wish to stop taking the drug.

### 3.2 Baseline Characteristics

The backgrounds of the 52 participants are shown in Table 2. No differences were observed in age, sex, height, weight, active stage, inflammatory range, treatment, or smoking status, while in terms of drinking status, there were more never drinkers in Group B.

**TABLE 2:** Participant background.

	Group A (*n* = 26)		Group B (*n* = 26)	
	FBRA → NF		NF → FBRA	
	(first)	(second)	(first)	(second)
Age (year)	40.7 ± 10.1		41.7 ± 10.8	
Sex (male, *n*)	14	(53.8%)	13	(50.0%)
Height (cm)	165.3 ± 9.3		163.4 ± 7.9	
Weight (kg)	57.8 ± 11.5		56.5 ± 11.0	
Active stage				
Remission (*n*)	7	(26.9%)	12	(46.2%)
Mild activity (*n*)	15	(57.7%)	8	(30.8%)
Moderate activity (*n*)	4	(15.4%)	6	(23.1%)
Type of UC				
Proctitis type (*n*)	10	(38.5%)	9	(34.6%)
Left‐sided type (*n*)	9	(34.6%)	9	(34.6%)
Total colitis type (*n*)	7	(26.9%)	8	(30.8%)
Treatment				
5‐ASA (*n*)				
Taking medication (*n*)	22	(84.6%)	21	(80.8%)
Not taking medication (*n*)	4	(15.4%)	5	(19.2%)
Smoking status				
Current smoker (*n*)	2	(7.7%)	1	(3.8%)
Ever smoker (*n*)	7	(26.9%)	8	(30.8%)
Never smoker (*n*)	17	(65.4%)	17	(65.4%)
Drinking status				
Daily (*n*)	5	(19.2%)	3	(11.5%)
Occasional drinker (*n*)	14	(53.8%)	11	(42.3%)
Ever‐drinker (*n*)	6	(23.1%)	2	(7.7%)
Never‐drinker (*n*)	1	(3.8%)	10	(38.5%)
Blood sampling at entry				
Total protein (g/dL)	7.53 ± 0.44		7.53 ± 0.50	
Albumin (g/dL)	4.51 ± 0.34		4.42 ± 0.31	
WBC (10 V3/mL)	5450 ± 17		5215 ± 11	
RBC (10 V4/mL)	452 ± 41		461 ± 41	
Hb (g/dL)	13.2 ± 2.2		13.5 ± 1.8	
Platelet (10 V4/mL)	27.8 ± 5.5		28.2 ± 9.8	
ESR 1 h (mm)	9.7 ± 9.0		8.7 ± 8.2	
ESR 2 h (mm)	23.1 ± 18.4		21.5 ± 17.4	
High sensitivity CRP (μg/L)	1057 ± 1461		1267 ± 2939	

Abbreviations: ESR, erythrocyte sedimentation rate; *n*, number; RBC, red blood cells, WBC, white blood cells.

### 3.3 Assessment of Primary Outcome

Table 3 shows the percentage improvement in the primary endpoint CAI. Six of the 26 patients (23.1%) in Group A (FBRA) and five of the 26 patients (19.2%) in Group B (NF) improved. The improvement in subjective symptoms at the third month after FBRA treatment was not significant.

**TABLE 3:** Improvement of clinical activity index (CAI).

First half	Group A		Group B		
	(FBRA) *n* = 26		(NF) *n* = 26		
Improved	6	(23.1%)	5	(19.2%)	*p* = 0.73
No change	5	(19.2%)	7	(26.9%)	
Worsened	15	(57.7%)	14	(53.8%)	

Second half	(NF) *n* = 20		(FBRA) *n* = 22		
Improved	10	(50.0%)	8	(36.4%)	
No change	8	(40.0%)	7	(31.8%)	
Worsened	2	(10.0%)	7	(31.8%)	
Did not attend second half	6	(−)	4	(−)	

Mix (first + second)	(FBRA *n* = 48)		(NF *n* = 46)		
Improved	14	(29.2%)	15	(32.6%)	
No change	12	(25.0%)	15	(32.6%)	
Worsened	22	(45.8%)	16	(34.8%)	

In the second half, eight of the 22 patients (36.4%) who received FBRA and 10 of the 20 patients (50.0%) who received NF showed improvement. In the first and second halves, 29.2% of the patients improved with FBRA and 32.6% improved with NF.

### 3.4 Assessment of Secondary Outcome

No serious adverse events leading to hospitalization or death were observed in either group.

In the ITT analysis, high‐sensitivity CRP, ESR (1‐h), white blood cell count, serum protein level, and serum albumin level all increased, especially high‐sensitivity CRP (17 of 26 patients (65.4%) in Group A and 10 of 26 patients (38.5%) in Group B), indicating that more patients in Group A improved than those in Group B. Improvement ratio 1.70; 95% CI 0.97–2.98 (Table 4).

**TABLE 4:** Improved blood data in the first half.

Intentional treaty analysis	Group A (FBRA)		Group B (NF)	
	*n* = 26		*n* = 26	
High sensitivity CRP (μg/L)				
Improved	17	(65.4%)	10	(38.5%)
No change/worsened	9	(34.6%)	16	(61.5%)
Improvement ratio (95% CI)	1.70	(0.97–2.98)	1	
ESR 1 h (mm)				
Improved	11	(42.3%)	8	(30.8%)
No change/worsened	15	(57.7%)	18	(69.2%)
Improvement ratio (95% CI)	1.38	(0.66–2.86)	1	
White blood cells (10 V3/mL)				
Improved	4	(15.4%)	2	(7.7%)
No change/worsened	22	(84.6%)	24	(92.3%)
Improvement ratio (95% CI)	2.00	(0.40–9.99)	1	
Total protein (g/dL)				
Increase	13	(50.0%)	8	(30.8%)
No change/decrease	13	(50.0%)	18	(69.2%)
Improvement ratio (95% CI)	1.63	(0.81–3.25)	1	
Albumin (g/dL)				
Increase	13	(50.0%)	10	(38.5%)
No change/decrease	13	(50.0%)	16	(61.5%)
Improvement ratio (95% CI)	1.30	(0.70–2.42)	1	

Analysis excluding cases of FBRA discontinuation	*n* = 20		*n* = 24	
High sensitivity CRP (μg/L)				
Improved	15	(75.0%)	10	(41.7%)
No change/worsened	5	(25.0%)	14	(58.3%)
Improvement ratio (95% CI)	1.80	(1.05–3.08)	1	
ESR 1 h (mm)				
Improved	11	(55.0%)	8	(33.3%)
No change/worsened	9	(45.0%)	16	(66.7%)
Improvement ratio (95% CI)	1.38	(0.66–2.86)	1	
WBC (10 V3/mL)				
Improved	4	(20.0%)	2	(8.3%)
No change/worsened	16	(80.0%)	22	(91.7%)
Improvement ratio (95% CI)	2.40	(0.49–11.77)	1	
Total protein (g/dL)				
Increase	10	(50.0%)	7	(29.2%)
No change/decrease	10	(50.0%)	17	(70.8%)
Improvement ratio (95% CI)	1.71	(0.80–3.67)	1	
Albumin (g/dL)				
Increase	10	(50.0%)	9	(37.5%)
No change/decrease	10	(50.0%)	15	(62.5%)
Improvement ratio (95% CI)	1.33	(0.68–2.62)	1	

Abbreviations: CRP, C‐reactive protein; ESR, erythrocyte sedimentation rate; WBC, white blood cells.

In the PPS analysis, which excluded eight patients who stopped taking the test food during the first half of the intervention period, 15 of 20 patients (75.0%) in Group A and 10 of 24 patients (41.7%) in Group B improved (improvement ratio 1.80; 95% CI 1.05–3.08). (Improvement ratio 1.80; 95% CI 1.05–3.08.) All other blood parameters also showed a trend toward improvement in Group A.

## 4 Discussion

In this double‐blind, randomized clinical trial of FBRA, FBRA did not significantly improve clinical symptoms. However, a greater proportion of patients who received FBRA improved high‐sensitivity CRP levels.

A randomized trial of a low‐fat, high‐fiber diet in UC patients in remission was reported [1]. In this trial, a low‐fat, high‐fiber diet improved the UC inflammation index. An open‐label clinical trial [6] on germinated barley foodstuffs for patients with UC in remission reported that germinated barley prolonged the remission period. The results of these previous studies support the results of our trial.

The strength of our trial is that it is a double‐blind study, unlike other clinical trials. Since UC is a disease that is often influenced by psychogenic factors and the primary endpoint of our trial is subjective symptoms, it is important to conduct a double‐blind study. Of note, limited clinical trials are conducted using dietary fiber for UC. In this study, we successfully created a placebo for FBRA, which has a similar appearance to FBRA and allowed us to conduct this study.

Unfortunately, no significant difference in subjective symptoms, the primary endpoint, could be demonstrated, but there was a trend toward improvement in subjective symptoms in the FBRA‐treated group. In the FBRA‐treated group, some subjects stopped taking the drug due to nausea or fullness; however, these symptoms were mild and disappeared after the drug suspension. No serious adverse events were observed. Therefore, the food was considered relatively safe.

A decrease in the amount of high‐sensitivity CRP, indicating inflammatory activity, was clearly observed in the population that received FBRA as scheduled. Other blood parameters on inflammatory activity also showed a trend toward improvement. These findings suggest that FBRA intake may improve UC.

## 5 Limitations

The limitations of this trial are as follows: (i) it was a single‐center study; (ii) the duration of FBRA administration was relatively short; and (iii) the sample size was relatively small. However, since this is an exploratory study and the results of this study showed the potential of FBRA in the treatment of UC, this trial could be used to plan other studies calculating the required number of patients, the dosage, and the duration of administration, and connected to a multicenter validation trial in the future.

## 6 Conclusions

Our double‐blind clinical trial showed there is an expectation that FBRA may be effective in improving inflammation in patients with UC. Therefore, a large‐scale validation study should be conducted in the future.

## Funding

This work was conducted with the financial support directly to the data center and test foods provided by Genmaikoso Co. Ltd. (Sapporo, Japan).

## Ethics Statement

This study was approved by the Ethical Review Committee of the Health Insurance Federation of Osaka Chuo Hospital in August 2006. The study registration number is UMIN000009364.

## Conflicts of Interest

The authors declare no conflicts of interest.

## Data Availability

The data that support the findings of this study are available on request from the corresponding author. The data are not publicly available due to privacy or ethical restrictions.
